# Two distinct olfactory bulb sublaminar networks involved in gamma and beta oscillation generation: a CSD study in the anesthetized rat

**DOI:** 10.3389/fncir.2014.00088

**Published:** 2014-07-30

**Authors:** Nicolas Fourcaud-Trocmé, Emmanuelle Courtiol, Nathalie Buonviso

**Affiliations:** ^1^Team Olfaction from Coding to Memory, Center for Research in Neuroscience of Lyon, CNRS UMR5292 - INSERM U1028Lyon, France; ^2^Team Olfaction from Coding to Memory, Center for Research in Neuroscience of Lyon, Université Claude Bernard Lyon 1Lyon, France

**Keywords:** olfactory bulb, oscillation, gamma, beta, current-source density, rat

## Abstract

A prominent feature of olfactory bulb (OB) dynamics is the expression of characteristic local field potential (LFP) rhythms, including a slow respiration-related rhythm and two fast alternating oscillatory rhythms, beta (15–30 Hz) and gamma (40–90 Hz). All of these rhythms are implicated in olfactory coding. Fast oscillatory rhythms are known to involve the mitral-granule cell loop. Although the underlying mechanisms of gamma oscillation have been studied, the origin of beta oscillation remains poorly understood. Whether these two different rhythms share the same underlying mechanism is unknown. This study uses a quantitative and detailed current-source density (CSD) analysis combined with multi-unit activity (MUA) recordings to shed light on this question in freely breathing anesthetized rats. In particular, we show that gamma oscillation generation involves mainly the upper half of the external plexiform layer (EPL) and superficial areas of granule cell layer (GRL). In contrast, the generation of beta oscillation involves the lower part of the EPL and deep granule cells. This differential involvement of sublaminar networks is neither dependent on odor quality nor on the precise frequency of the fast oscillation under study. Overall, this study demonstrates a functional sublaminar organization of the rat OB, which is supported by previous anatomical findings.

## Introduction

Odor coding in the first cortical relay of the olfactory system has been shown to have both spatial and temporal components. In the mammalian olfactory bulb (OB), three types of oscillatory rhythms have been described *in vivo* (for a review see Kay, 2014). The first is a slow oscillation, in the theta frequency range, related to the animal respiration’s rhythm (for a review see Buonviso et al., 2006). This slow rhythm is particularly important because it sets both the rate and intensity of stimulus sampling and modulates the detection threshold (Kepecs et al., 2007; Carey and Wachowiak, 2011; Courtiol et al., 2011; Esclassan et al., 2012). In addition, it plays a role in pacing odor representation, whereby each cycle contains the entirety of the stimulus information (Kepecs et al., 2006; Schaefer and Margrie, 2007) or even modulates the code according to its frequency (Verhagen et al., 2007). Superimposed on this slow rhythm, faster oscillations in the gamma (40–90 Hz) and beta (15–30 Hz) ranges have been recorded in both conscious (Freeman and Schneider, 1982; Gray and Skinner, 1988; Kay, 2003; Ravel et al., 2003) and anesthetized animals (Buonviso et al., 2003; Neville and Haberly, 2003). *In vivo* experiments have shown that these fast rhythms are modulated by animal experience and task (Ravel et al., 2003; Martin et al., 2004; Beshel et al., 2007; Kay and Beshel, 2010), suggesting that fast oscillatory rhythms are involved in odor coding. Whereas gamma oscillations have been identified as necessary for fine odor discrimination (Nusser et al., 2001), beta oscillations seem to be related to odor discrimination learning (Martin et al., 2006; Kay and Beshel, 2010; for review, see Kay, 2014).

Regarding the generation of the two fast oscillations, although theoretical studies (Rall and Shepherd, 1968) and *in vitro* (Lagier et al., 2004) and *in vivo* (Lepousez and Lledo, 2013) experiments emphasized the role of the mitral-granule loop through their dendro-dendritic synapses for generating the gamma rhythm, the origin of the beta rhythm is poorly understood. Beta rhythm has been only observed *in vivo* and is abolished when centrifugal feedback to the OB is disrupted (Neville and Haberly, 2003; Martin et al., 2006). A qualitative current-source density (CSD) analysis in anesthetized and tracheotomized rats (Neville and Haberly, 2003) shows that gamma and beta rhythms could be evoked by different odor concentrations. Neville and Haberly’s study suggested that beta and gamma rhythms are generated by overlapping networks in the OB, but this study: (1) only showed a limited duration example of CSD for gamma and beta oscillations without further detailed analyses, such as either taking into account cycle-to-cycle variability or the dependence of CSD on OB stimulation or on the oscillation frequency; and (2) was not performed under basal conditions (i.e., the rats were tracheotomized and odorants were constantly aspirated for a long duration).

CSD (Mitzdorf, 1985; Pettersen et al., 2006) analysis is an efficient method for uncovering the spatial location of extracellular current sinks and sources that generate the local field potential (LFP) during neural network activity. Combined with the extensive knowledge of bulbar network anatomy (see Ennis et al., 2007 for a review), CSD analysis allows a better understanding of the neuronal population involved in generating field potential dynamics. Thus, the aims of the present study were to study the OB network implicated in the generation of fast beta and gamma oscillations using: (1) the CSD method in a more quantitative and detailed manner than in previous studies; and (2) a more physiological model of freely breathing anesthetized rats in which beta and gamma oscillations can alternate in response to the same odorant stimulation.

## Materials and methods

### Preparation and electrical recordings

**Figure 1:**
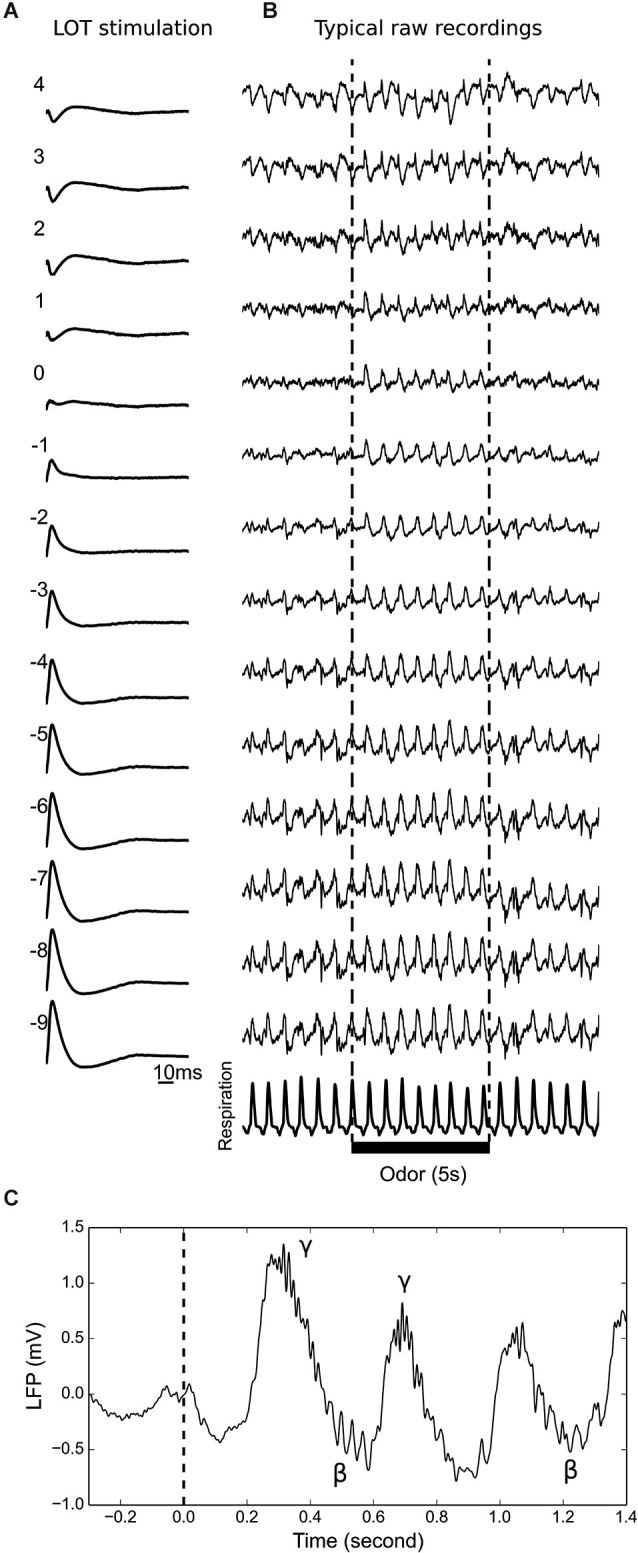
**Determination of the mitral cell layer and multielectrode recording examples. (A)** Typical recordings at 14 locations along the silicon probe during electrical LOT stimulation. The electrode closest to the signal inversion is numbered 0, which is consequently designated as the closest to the MCL. The EPL and GL are above, and the GRL is below. **(B)** Typical 15 s recording displayed together with the respiratory signal. Odor presentation is indicated by the black bar. During the SPONT period, there was a slow and large amplitude LFP oscillatory rhythm that reversed at the MCL. During the ODOR period, the slow oscillation reversed close to the GL. **(C)** Example of an LFP trace showing the alternation of gamma (γ) and beta (β) oscillations on top of the slow rhythm (linked to respiration) at the beginning of the ODOR period (dashed line).

Male Wistar rats (150–350 g) obtained from Charles River Laboratories (L’Arbresle, France) were anesthetized with urethane (i.p. 1.5 mg/kg, with additional supplements as needed) and placed in a stereotaxic apparatus. All experiments were performed in accordance with the guidelines of the European Communities Council. The dorsal region of the OB was exposed. Bulbar activity was recorded as a broadband signal (0.1–5 kHz) using linear 16-channel silicon probes (NeuroNexus Technologies, Ann Arbor, MI) with a homemade, 16-channel DC amplifier. Electrodes on the probe were 50 μm apart (or 100 μm, in the sole case of Figure 1C). The data were digitally sampled at 10 kHz and acquired on a PC using the IOTech acquisition system (Wavebook, IOTech Inc., Cleveland, OH). The respiration signal was recorded using a homemade flow-meter based on a fast response time thermo-dilution airflow sensor. Odors were delivered through a dilution olfactometer (440 ml/min). The recording protocol was as follows: 5 s of spontaneous activity (SPONT), 5 s of odor-evoked activity (ODOR), and 5 s of post-stimulus activity. The odors used were heptanol (A07), ethyl-heptanoate (E07), 2-heptanone (K07), isoamyl-acetate (ISO), and heptanal (D07), each at 10% of the saturated vapor pressure. The time delay between each odor presentation was at least 1 min.

### Lateral olfactory tract (LOT) stimulation and mitral cell layer determination

Bipolar stainless steel electrodes were positioned on the lateral olfactory tract (LOT; coordinates relative to bregma: AP +3.7 mm, L 3.4 mm). Optimal placement was determined by the observation of field potentials evoked in the OB by electrical stimulation (constant current square pulses of 200 μs with an amplitude range of 0.1–0.5 mA).

At each recording location, the recording probe was advanced into the ventral OB while monitoring the spontaneous LFP activity and the response to LOT stimulation. The advancement was stopped when the mitral cell layer (MCL) was between electrodes 4 and 8 (electrode 1 was close to the glomerular layer (GL)). In some experiments, only signals from 14 adjacent electrodes of a given probe were recorded. Precise determination of the MCL location was performed off-line (see Figure 1). After MCL determination, electrodes of a given recording location were reindexed for analyses with electrode index 0 as the closest to the MCL, positive electrode indices towards the external plexiform layer (EPL) and GL, and negative electrode indices towards the granule cell layer (GRL; see Figure 1 for an example).

### Oscillation detection

Transient oscillatory events in the LFP signals were detected using Openelectrophy software^1^ (Garcia and Fourcaud-Trocmé, 2009). This software program uses the methods described by Roux et al. (2007). The method consists of computing the time frequency power map of the signal using a standard Morlet wavelet transform and detecting hot spots due to oscillatory events by thresholding the map. The threshold was different for each frequency range of interest. At the lower range, for the slow oscillation linked to respiration, the threshold was adjusted manually at each recording site to ensure that the slow oscillation was detected both before and during odor presentation. For the beta and gamma ranges, oscillatory events only occur during odor presentation in anesthetized rats (Buonviso et al., 2003); thus, the threshold was computed as the mean +/− *N* standard deviations of the time frequency power map during SPONT recording in the same frequency range (gamma range *N* = 8, beta range *N* = 3). The ridge from each detected event was extracted on the time frequency power map, which determined the time-frequency course of each transient oscillatory event, together with its instantaneous phase and energy. Only oscillatory events lasting more than three cycles were kept for further analysis. Finally, each oscillation was visually checked, and artifact-detected events produced by a single large fluctuation of the LFP were removed (95 out of 841 oscillations in the beta range).

### CSD computation, averaging across oscillation cycles and recording locations

The layered and approximately spherical organization of the OB allows the computation of the CSD in one dimension (Rall and Shepherd, 1968). For each recording site, the CSD maps were computed with the inverse current-source density method (iCSD, Pettersen et al., 2006). Compared with standard methods, which require an estimate of the LFP spatial second derivative (Mitzdorf, 1985), the iCSD method computes a model of the LFP assuming an arbitrary distribution of sources or sinks across electrodes and then reverses the model to obtain the estimated sources and sinks from the actual data. This process accounts for the long-range influence of sinks and sources and enables the acquisition of a CSD estimation for every electrode, including electrodes at the ends of the silicon probe. To attenuate signal variability, sinks and sources were smoothed by computing the CSD and then spatially averaging over three electrodes, which produced very similar results to a standard CSD method that computes the spatial second derivative of the raw signal. Following standard assumptions (see Rall and Shepherd, 1968, for a discussion), we considered the tissue resistivity to be the same throughout and assumed that average extracellular currents flowed parallel to the probe. As the tissue resistivity was unknown, the CSD was expressed in arbitrary units (a.u.). Finally, the CSD was generally computed for a given frequency band only (except in Figure 8, left panels). This calculation was achieved by filtering the raw data prior to computing the CSD or performing any averaging (fast Fourier transform-filtering performed on the whole 15-s trial).

**Figure 2:**
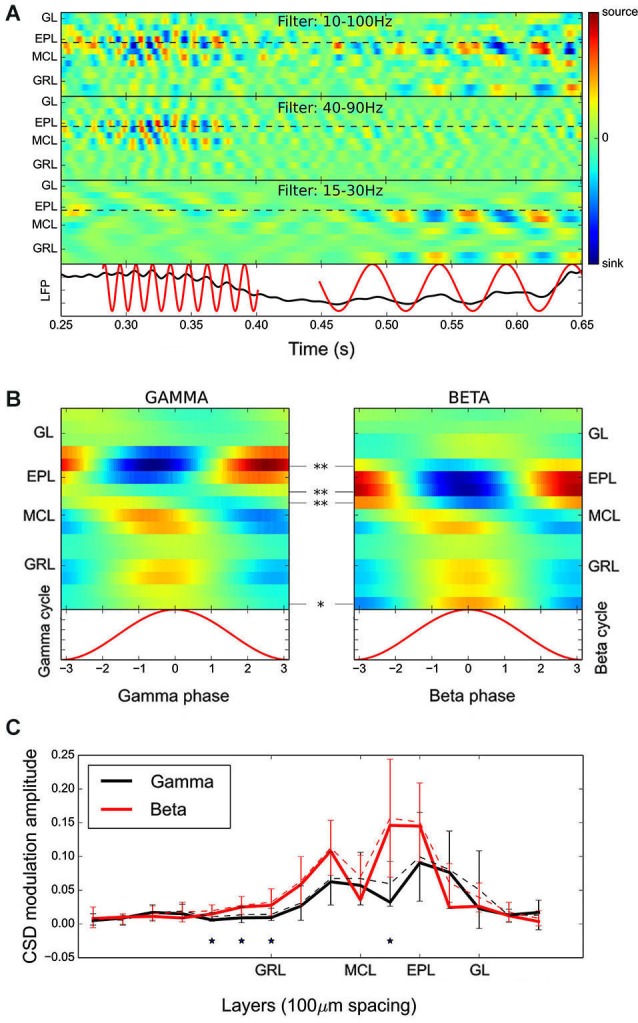
**CSD analyses of fast rhythms revealed that different sub-layers of the OB network are involved in beta and gamma oscillation generation. (A)** Time evolution of the CSD for a single multielectrode recording filtered in three different frequency bands (from top to bottom: 10–100 Hz, 40–90 Hz, and 15–30 Hz). The plotted time represents a single respiratory cycle. Below is the raw signal recorded from the deepest electrode in the GRL, on which are superimposed the detected gamma and beta oscillations. Visual comparison of the different CSD maps (use the black dashed lines as a guide) shows that the major current sources and sinks during beta and gamma oscillations appear at different layers, spaced by one to two electrodes, i.e., 50–100 μm. **(B)** Time evolution of the group average CSD maps across the gamma (left) and beta (right) cycles. Statistical significances are given as an electrode-by-electrode CSD amplitude comparison (range of recording sites averaged per electrode: *N* = 9–18). The current sink in the EPL was present in more superficial layers during the gamma oscillation. **(C)** Average amplitude of sink/source oscillation at each electrode during gamma (black) and beta (red) oscillations (error bars indicate the standard deviations). These data were obtained with electrodes spaced at 100-μm intervals. The results confirm the spatial shift between gamma and beta current sources/sinks observed in (**A** and **B**) and additionally show that the beta oscillation is involved in a larger zone of the GRL. Statistical significances are assessed as in **(B)**. Note that the solid line corresponds to the amplitude of the group CSD modulation and the dashed line is the average of individual CSD amplitude modulation, on which statistical testing was performed.

**Figure 3:**
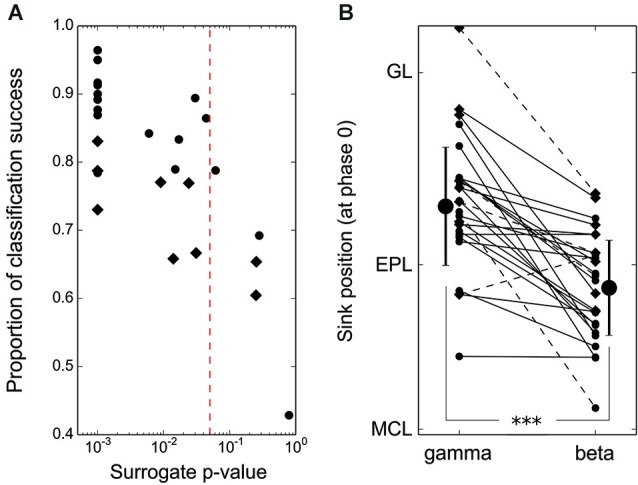
**Spatial shift of beta and gamma EPL activation is significant at the single recording site level. (A)** Results of the leave-one-out classification test performed individually at each recording site (50-μm spaced electrodes: round markers, 100-μm spaced electrodes: diamond markers). The *p*-values reported on the x-axis were obtained by comparing single recording site actual data with 1000 randomizations of gamma/beta labels of every CSD oscillation map from the same recording site. The vertical red line indicates the 0.05 significance threshold. Note that 100-μm spaced electrodes most often have significant beta/gamma classifications but with lower classification rate success due to poorer spatial resolution. **(B)** Position of the EPL sink at phase 0 for each individual CSD map (average across recordings from a single recording site location) during the beta vs. gamma oscillations. Markers as in **(A)**, dashed lines correspond to cells with a non-significant beta/gamma classification test in **(A)**. The latter are not taken into account for group averages and standard deviations (large markers).

**Figure 4:**
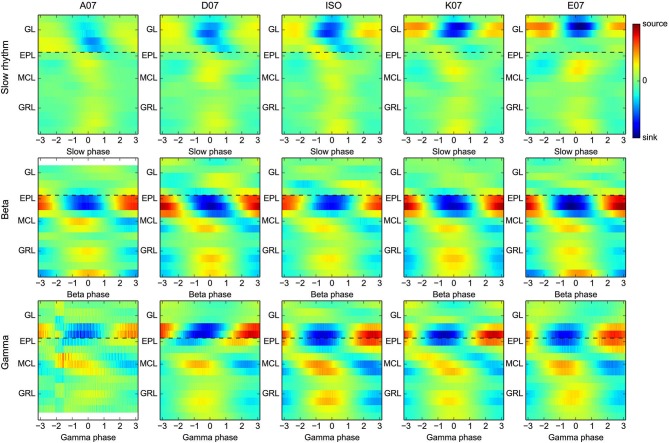
**Asymmetrical and differential activation of the glomerulus map by distinct odors does not affect the time evolution and spatial organization of beta/gamma CSD maps.** Group average CSD maps were computed for slow oscillation and beta and gamma rhythms (from top to bottom, respectively) separately for each odor used (each plot column corresponds to a single odor). The color scales are identical across either all slow oscillation maps or all fast (gamma and beta) oscillation maps. Black dashed lines are only plotted to provide a visual guide. Note all 18 recording sites have been stimulated with all five odors, but gamma or beta oscillations were not always generated and detected. Thus, slow oscillation group CSD maps are averages across the 18 sites, whereas gamma and beta maps are averages of (in plot order) *N* = 10, 12, 12, 13, 14 and *N* = 3, 17, 13, 18, 18, respectively.

**Figure 5:**
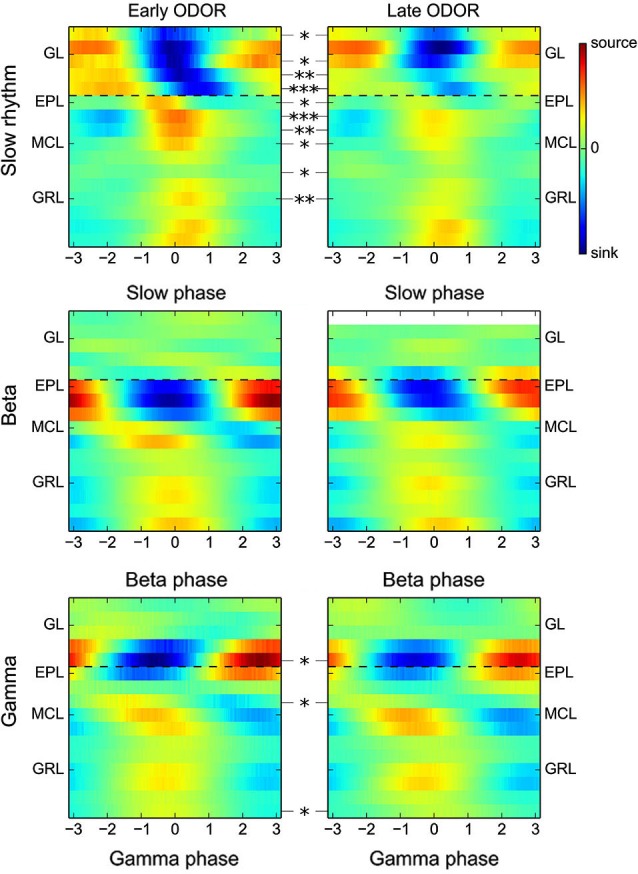
**Stability of gamma and beta CSD maps over the course of odor presentation.** Group CSD maps computed for either the slow oscillations (range 0.5–5 Hz, top panel), beta oscillations (middle) or gamma oscillations (bottom) at the beginning of the ODOR period (0–1 s, left) or at the end of the ODOR period (3–5 s, right). Statistical analyses of the CSD map amplitude show a marked decrease of the CSD modulation amplitude in the MCL, EPL and GL layers for the slow oscillation, but no or very few significant changes for beta and gamma oscillations. In addition, the spatial organization of fast oscillation CSD maps is unchanged between the early and late ODOR period (dashed black lines are visual guides).

**Figure 6:**
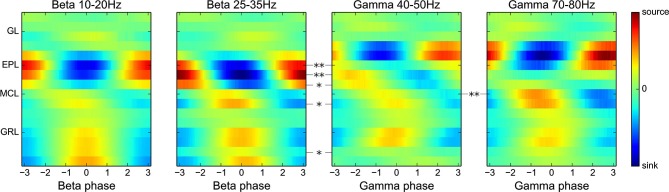
**Oscillation frequency is not the main determinant for CSD map spatial organization.** Group average CSD maps have been computed for the detected oscillations in either the low or high beta range (10–20 Hz or 25–35 Hz, respectively) and oscillations in either the low or high gamma range (40–50 Hz or 70–80 Hz, respectively). Most significant differences (assessed by an electrode-by-electrode modulation amplitude comparison) are found between the beta and gamma maps and not between the low and high frequency range maps for the same oscillation type (in plot order: *N* = 16, 14, 11, 18). The color scales are distinct for the beta and gamma maps for better visualization.

**Figure 7:**
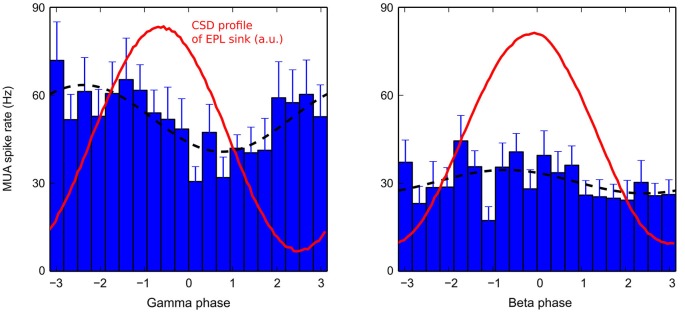
**Average MUA in the MCL during a gamma (left) or beta (right) cycle**. Average MUAs (bar plots) during gamma (left) or beta (right) oscillations are fitted with a sine function (black dashed lines). To compare with the corresponding CSD dynamics in the EPL, the time evolution of EPL sinks (taken from electrode 4 for gamma, and from electrode 2 for beta, see Figure 2B) are plotted in red (red curve maxima correspond to the deepest EPL sinks in blue in Figure 2B). MUAs were first computed for each recording sites, then averaged across recording sites for plotting. Error bars are +/− SEM (gamma: *N* = 18, beta: *N* = 17).

**Figure 8:**
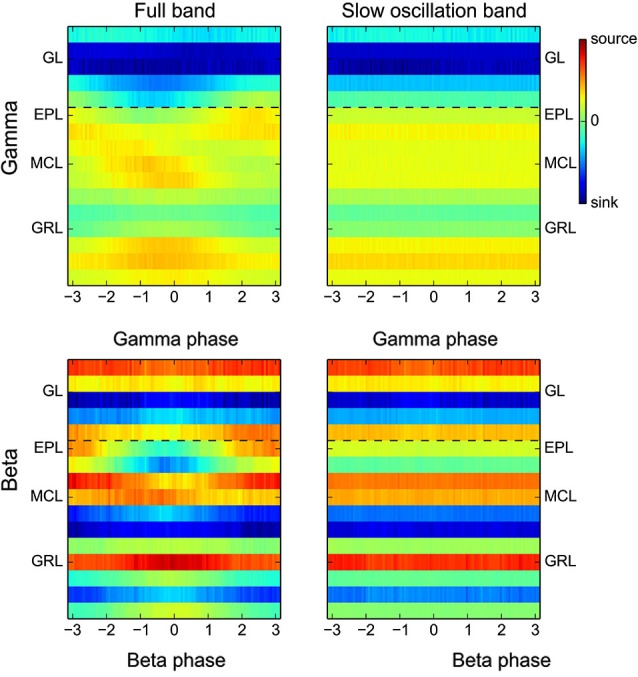
**Full band CSD during gamma or beta oscillations.** Group CSD maps computed as in Figure 2B but either without prior filtering of the raw signal (full band, left panels) or with a filtering of the raw signal in the low frequency band only (0.1–10 Hz, right panels). The slow CSD modulation adds large and constant offsets, different for each electrode, to the fast CSD modulation. Right panels display only the offsets while left panels display the addition of slow and fast CSD modulations. Due to the additivity of the slow and fast CSD modulations, the difference between left and right panels is qualitatively equivalent to Figure 2B.

Most of the figures display a time-space map that shows the temporal evolution of the one-dimensional CSD across one typical oscillation cycle. For each recording location and frequency range, one individual CSD map was computed first by averaging the filtered electrode signals across oscillation cycles in the corresponding frequency range and then by applying the iCSD algorithm. A group CSD map was then obtained by averaging individual CSD maps from distinct recording locations. The average across oscillation cycles was simply calculated by aligning the time points corresponding to the same oscillation phase. Time points were grouped in 100 bins per cycle, and oscillation phases plotted in the figure x-axes ranged from −3.14 to 3.14 (in radians), spanning a whole single oscillation cycle. The number of averaged cycles depended on the location and the frequency-band of interest. In fact, the number of odor presentations could vary between recording locations, and for a given odor, gamma or beta oscillation expression could also vary. Thus, the number of averaged cycles ranged across locations in the following manner: from 50 to 640 cycles in the gamma band, from 12 to 135 cycles in the beta band, and from 27 to 198 cycles in the slow oscillation band. To average across recording locations, the individual CSD maps were spatially shifted to align the locations of the MCL. The number of averaged recording locations (for electrodes spaced 50 μm apart) was 18, although one location displayed no detectable beta oscillations. Only layers for which at least 40% of the individual maps were averaged were used for plots and analyses.

### CSD map statistical comparisons and classification

Group CSD maps were averaged across recording locations. To compare group CSD maps under different conditions, the analysis used individual CSD maps from which amplitudes of the source-sink modulation (the distance from CSD minimum to maximum values) were computed for each electrode. Groups of CSD modulation amplitudes from the same electrode (relative to the MCL location) of different recording sites were then compared by an appropriate paired or unpaired Wilcoxon test. Throughout the figures, *p*-values are given by *p* < 0.05 (*), *p* < 0.01(**), and *p* < 0.001 (***). Because the maps had to be aligned according to the location of the MCL, the averages of the CSD maps at electrodes far from the MCL were generally calculated only on a subset of the recording locations. The minimal proportion of recording locations that could be averaged, which was necessary for an electrode to be taken into account for the plots and statistical analyses, was 40% of all recording locations. The final ranges of averaged locations are given in the text. Importantly, estimating the amplitude of an electrode CSD modulation is not a linear operation. Due to the jitter of modulation phases across recording locations, the amplitude of the averaged map is always lower than the average of the individual map amplitudes. Statistical calculations were performed on the latter, whereas the former was closer to the group CSD maps shown in the figures.

A classification test was used to ensure that the observed differences between the gamma and beta CSD patterns not only represent a group effect but are still valid at individual recording sites. For a given recording site, the CSD maps were computed for each detected oscillatory event in either the gamma or beta range (average across cycles, 20 points per cycle). Then, a leave-one-out classification test was applied to the CSD map ensemble from this site. Basically, each oscillation map was classified based on its Euclidean distance from the beta or gamma average maps (computed with the remaining single oscillation maps of the recording site). The percentage of correct classifications was compared with the distribution of percentages computed in the same manner, but only after shuffling all single oscillation maps (1000 shuffles). Note that for this classification test, single oscillation maps were z-scored to compare CSD patterns only, by attenuating differences in oscillation amplitudes.

An estimation of the positions of sink and source maximal amplitude was performed at phase 0 of the beta or gamma oscillations. Individual CSD map values at phase 0 were interpolated with a spline of degree 4. The maximal sink amplitude was detected as the interpolated curve maximum between 0 and 300 μm above the MCL, and the maximal source amplitude was detected as the curve minimum between −200 and 0 μm below the MCL.

### Mitral multiunit activity detection

For spike multiunit activity (MUA) detection, all recordings (regardless of the odor stimulus or experimental condition) from a given electrode at a given location were concatenated, and this raw signal was high-pass filtered above 300 Hz. Spikes were then detected as short events with peaks that were more than seven standard deviations from the signal mean and that had an inter-spike interval > 2 ms. The MUA pooled from electrodes −1 to 1 (0 was assigned to the electrode closest to the MCL) was considered representative of mitral cell spiking activity.

## Results

Data were recorded from 18 locations in 12 anesthetized rats (one or two locations per rat) situated in the ventral half of the OB. The probe was oriented perpendicularly to the OB layers to measure the LFP in all layers simultaneously. At each recording location, the electrode closest to the MCL was determined by a polarity reversal of the bulbar potential evoked by electrical stimulation of the LOT (see Rall and Shepherd, 1968, and Figure 1A). The critical recording depths were the MCL, the GL, the EPL, and the GRL, as defined by Rall and Shepherd (1968).

After placement of the probe, the rat was stimulated with a set of odorants chosen for their ability to evoke different oscillatory activity patterns (Cenier et al., 2008). A typical recording consisted of the following three 5-s periods: spontaneous activity (SPONT), odor presentation (ODOR), and post-stimulation activity (Figure 1B). In this study, only the SPONT and ODOR periods were analyzed.

To take advantage of the laminar and symmetrical organization of the OB, a one-dimensional CSD analysis was performed using the iCSD method (Pettersen et al., 2006, see Section Materials and Methods for additional details). For the quantitative analysis of the CSD during oscillations, the oscillation instantaneous phases were automatically detected using a wavelet method. This method allowed us to average the raw signals across oscillation cycles and to compute the CSD spatio-temporal map during a single typical cycle. Note that the oscillations used as a reference for cycle averaging were taken from the deepest electrode in the GRL, where oscillations were the most stable across recording locations. These individual CSD maps, computed for each recording location, were then further averaged across different recording locations after an appropriate spatial shift to align the electrodes closest to the MCL (see Section Materials and Methods).

### Different sublaminar networks are involved in gamma and beta oscillation generation: the gamma rhythm involves more superficial layers than the beta rhythm

As previously observed (Buonviso et al., 2003; Neville and Haberly, 2003; Cenier et al., 2008; Fourcaud-Trocmé et al., 2011), odor presentation elicited fast LFP oscillations in the gamma (40–90 Hz) and/or beta range (15–30 Hz), superimposed on a slow rhythm (0.5–5 Hz) with a large amplitude that was linked to rat respiration. Gamma oscillations were locked to the inspiration-expiration transition, and beta activity usually occurred during late expiration (see Figures 1C and 2A for examples, and Buonviso et al., 2003, for a more complete description of the gamma/beta alternation in the freely breathing anesthetized rat).

Figure 2A shows an example of the time evolution of the CSD computed with a single recording along with the raw signal recorded from the deepest electrode in the GRL. Beta and gamma oscillations are clearly visible on the raw trace and the corresponding alternation of sources and sinks in the CSD can be observed when the CSD is computed in the appropriate frequency band (see top panels in Figure 2A). Note that in this study, the slow signal component was removed due to the high-pass filtering. Thus, the fast oscillation current sources and sinks displayed in the figures are only fast modulations around the ongoing CSD slow modulation. As previously stated, gamma and beta oscillations were automatically detected on signal from the deepest electrode in the GRL (see lower panel in Figure 2A, red traces), and group CSD maps were computed (Figure 2B).

The gamma CSD map showed a clear dipole between the EPL and the MCL/GRL, with a polarity that alternated over time (see Figure 2B, left panel), and the GL was not involved in this process. This finding confirms previous results (Neville and Haberly, 2003). Above the MCL, the CSD oscillation amplitude was maximal in the upper EPL (electrode 4, with 0 located near the MCL, i.e., approximately 200 μm above the MCL). Below the MCL, two sources are visible. One was close to the MCL (electrodes −1 and 0), and the other was deeper in the GRL (electrode −5).

Contrary to previous observations (Neville and Haberly, 2003), the beta CSD map (Figure 2B, right panel) differed substantially from that of the gamma oscillation. First, above the MCL, the beta current source/sink oscillation amplitude was maximal in the lower EPL (electrode 2, approximately 100 μm below the gamma maximal source/sink modulation) and similar in amplitude to that observed during gamma oscillation; in fact, the maximum modulation amplitude in the EPL was 0.064 a.u. for the beta CSD map and 0.068 a.u. for the gamma CSD map. An electrode-by-electrode statistical analysis (Figure 2B, see Section Materials and Methods) confirmed the existence of a significant difference in CSD amplitude modulation as a function of the layers between the gamma and beta CSD maps. Interestingly, during beta oscillations, the CSD analysis showed a pronounced oscillatory current source locked to the LFP in the deep GRL layers (electrodes <−5), which was absent during the gamma oscillation. To investigate this difference and confirm that it was not an edge effect in the CSD map estimation, additional recordings (*N* = 10 recording sites in 6 additional rats) using silicon probes with electrodes spaced by 100-μm intervals were performed. This process allowed the exploration of a greater bulbar depth, but at the expense of spatial resolution. The CSD modulation amplitude (average and SD) obtained with these probes at each electrode is shown in Figure 2C. It displayed the same shift in the spatial organization of current sinks and sources as in Figure 2B and confirmed that the deep GRL (300–500 μm from the MCL) was significantly more activated during beta oscillations.

In most of the individual recordings, the different locations for the beta/gamma CSD modulation in the EPL could be directly observed (compare the different panels of Figure 2A). However, due to the variability between recording locations, it was necessary to quantitatively assess the difference between gamma and beta CSD maps at each individual recording site, which was performed in two steps. First, a classification test was used to demonstrate that beta and gamma CSD maps were indeed distinguishable at the single site level. Basically, for each site, one CSD map was computed for each detected oscillatory event, and a leave-one-out classification test was then performed on these maps to estimate the proportion of maps correctly classified as beta or gamma (see Section Materials and Methods). Figure 3A shows the proportion of correct classifications among single oscillation CSD maps at each site (except two sites where no beta oscillation was detected) vs. a statistical *p*-value assessed by a shuffling method of single oscillation CSD maps (see Section Materials and Methods). For more than 80% of the recording sites (21 out of 26 recording sites), single oscillation gamma and beta CSD maps were reliably classified with *p* < 0.05 (Figure 3A, significance threshold is the red dashed line). Note that *p* = 0.001 was the minimum possible value due to the 1000 shuffles used to compute the surrogate data (Figure 3A). In a second step, determining that the significant differences found in the classification test were indeed the same that were found at the group level was necessary. The comparison was performed by determining the position of the maximal sink amplitude in the EPL at phase 0 (see Section Materials and Methods). The results, shown in Figure 3B, were in good agreement with the group results; at most recording sites, the gamma EPL sink was more superficial than the beta EPL sink. The average EPL sink distances from the MCL were 203 μm and 128 μm for gamma and beta, respectively (paired Wilcoxon test *p* < 0.001, *N* = 21). Similarly, below the MCL, the maximal source amplitude at phase 0 was slightly but significantly shifted between the gamma and beta rhythms; the average source distances from the MCL were −45 μm and −70 μm for gamma and beta, respectively (paired Wilcoxon test *p* < 0.01, *N* = 21).

Overall, these analyses show that gamma oscillation involves more superficial layers of the EPL than beta oscillation. This observation is also true in the GRL, which shows a smaller shift in the CSD modulation peak location, but a larger extent of the deep GRL is involved in the beta oscillation.

### The differential sublaminar CSD profile of beta and gamma oscillations does not depend on odor quality

An important assumption of CSD computation is that bulk extracellular current flows are parallel to the electrode, which is due in particular to the laminar and symmetrical organization of the OB. However, this approximation is clearly violated during odor presentation. Indeed, different odors activate different sparse spatial patterns of glomeruli at the surface of the OB. This outcome necessarily results in a relatively asymmetric activation of each OB layer, even if activity redistribution can occur through lateral interactions. Due to these limitations, it was important to verify whether the previous results pertaining to the spatial organization of gamma and beta oscillation hold true regardless of the odor presented. As a control, CSD maps have also been computed for slow oscillations linked to the respiratory rhythm (0.5–5 Hz range, clearly visible in the example shown in Figure 1B), which was achieved by computing group CSD maps for each oscillation type, as in Figure 2B (slow oscillation, beta, and gamma), but separately as a function of the odor used. Group maps for each oscillation type and odor are shown in Figure 4. For the slow oscillation (Figure 4, top), there is a clear odor-related difference between the group CSD maps, which is likely due to the differential activation of the OB by each odorant, particularly the strength of the glomerulus activation and the generated slow depolarization in mitral cells (Cang and Isaacson, 2003; Briffaud et al., 2012). However, for fast oscillations, a systematic comparison of group CSD maps by odor pairs showed that no (and, in a very few cases, one) significant difference was found when comparing two gamma CSD maps or two beta CSD maps; the CSD maps of both gamma and beta oscillations are thus stable regardless of the odor. In contrast, two or more significant differences were systematically found when comparing the beta and gamma CSD maps evoked by the same odor, with the exception of odor A07, due to the very small number of recording sites displaying gamma oscillations with this odor (*N = 3*); the differential CSD profile of beta and gamma oscillations is thus persistent across different stimuli. All odors displayed the same spatial shift of EPL maximal CSD modulation as previously observed when all odor recordings were taken together (compare Figures 4 and 2B). The same analysis on data from electrodes with 100-μm spacing confirmed the larger sink/source modulation amplitude in the deep GRL during beta oscillation for all odors (data not shown).

Interestingly, the amplitude of the first LFP slow oscillation cycle following the beginning of odor presentation was often observed to be higher and generated larger amplitude sink-source modulation than during the following cycles (see Figures 1C and 5, top panels). Particularly, slow oscillation group CSD maps involved a GL-MCL sink-source dipole at the inspiration-expiration transition (phase 0) due to the excitation of mitral cells by olfactory nerve inputs and with an amplitude that is significantly larger in the early part of odor stimulation (0–1 s after odor presentation). The decrease in the late odor stimulation (3–5 s after odor presentation) is likely due to a global adaptation of the early olfactory system to the odor input. To verify whether this effect has an impact on gamma or beta group CSD maps, fast oscillations were grouped according to their detection time (early or late odor presentation times: 0–1 s and 3–5 s, respectively). Corresponding group CSD maps did not show any differences in spatial organization (identical to the initial group CSD maps) and only minor significant differences in amplitude (Figure 5, bottom panels). Overall, these analyses show that the differential laminar CSD profile of beta and gamma oscillations is neither affected by the quality of OB input nor by the amplitude of the LFP slow oscillation.

### The differential sublaminar CSD profile of beta and gamma oscillations does not depend on precise oscillation frequency

The neuronal membrane is known to act as a low pass filter of intracellular currents. Recent modeling studies (Łęski et al., 2013) have demonstrated that this action affects the extent of dipoles generated by rhythmic synaptic activation, so that faster rhythmic activation of the same set of synapses should generate a smaller dipole. Consequently, the extent and location of a fast oscillation sink/source pair should vary continuously with oscillation frequency. Here, the occurrence of such an effect would explain why the gamma rhythm CSD showed a lower extent with a closer sink/source dipole from the upper EPL to the MCL whereas the major beta dipole extended from the lower EPL to the deep GRL. To confirm such a caveat, group CSD maps were computed separately for the low and high portions of the beta and gamma frequency ranges. To obtain a clear separation, frequency ranges were extended to 10–20 Hz and 25–35 Hz for low and high beta oscillations, respectively. Gamma frequencies were separated between 40–50 Hz and 70–80 Hz for low and high gamma oscillations, respectively. Group CSD maps corresponding to these frequency ranges are displayed in Figure 6. Between the low and high gamma ranges, except for a small increase in the CSD modulation amplitude in the MCL, which may indicate a larger MCL activation, no significant differences were found. Similarly, no significant differences were found between the low and high beta CSD maps. However, high beta vs. low gamma CSD maps showed significant differences similar to those obtained previously with the full frequency range of beta and gamma oscillations (Figure 2B). The same analysis performed with data from 100-μm spaced electrodes confirmed that the significant largest activation of deep granule cells is obtained for beta oscillations and is not a frequency effect (data not shown).

Overall, these results eliminate the possibility that the source/sink differences between beta and gamma CSD maps could be an effect of oscillation frequency and show that there is a clear-cut distinction between the beta and gamma CSD profiles.

### Mitral firing can explain the observed CSD map modulations

To better understand the origin of the sink/source modulation during fast rhythms, MCL MUA was detected (see Section Materials and Methods). The time-evolution of the average MCL MUA firing rate during gamma or beta oscillation is shown in Figure 7. In both cases, the MUA activity was phase-locked to the fast oscillations (Rayleigh test, *p* < 10^−4^). However, the MUA modulation was strong during gamma oscillations and weak during beta oscillations (amplitude of MUA modulation sine fit: for gamma: 52.4 +/− 11.6 Hz; for beta: 35.0 +/− 5.0 Hz, black dashed lines in Figure 7, see Cenier et al., 2009 for a more extensive study of this point). Interestingly, for both rhythms, the peak of MUA activity occurred before the sink maximum in the EPL layer. Assuming a typical gamma oscillation of approximately 60 Hz and a beta oscillation of approximately 22 Hz, the time advance of the MUA peak relative to the corresponding CSD sink in the EPL (maximum of red curve in Figure 7) was on the order of 4.5 ms during gamma oscillation and 6.3 ms during beta oscillation. Both measures are compatible with the time needed for spike propagation in the mitral cell lateral dendrites and activation of fast AMPA synapses onto granule cells (generating the EPL sink). Thus, this finding supports the idea that a large fraction of the CSD modulation during fast rhythms is due to granule cell excitation by mitral cells. Granule cell feedback inhibition onto mitral cells is likely another component of the CSD modulation; however, there is, in this preparation, no reliable estimation of mitral inhibition dynamics. An *in vitro* study showed that mitral IPSPs are phase-locked to gamma oscillations but in anti-phase relative to mitral spikes (see Lagier et al., 2004, their Figures 4E,G). In the current CSD interpretation, this effect would reinforce the EPL current source observed around the phase +/−π. Furthermore, because inhibition has a slowest time constant than excitation, inhibition-related CSD modulation is more filtered by beta and gamma fast oscillations than the excitation-related component. Overall, this finding demonstrates that gamma and beta CSD dynamics may be explained by the rhythmic excitation of granule cells by mitral cells and possibly enhanced by the delayed inhibitory feedback by granule cells onto mitral cells.

### Relationship between fast and slow CSD modulations

Up to this point, in order to study how CSD maps were modulated by the fast LFP oscillations, raw signals were initially filtered in the frequency band of interest (Figures 2A,B). However, these fast oscillations are superimposed on the slow rhythm driven by the animal respiration (Figure 1C). The CSD modulations generated by this slow rhythm (see Figures 4 and 5 top rows) are about 10 times larger and are superimposed to the fast CSD modulations generated by beta or gamma rhythms. To have a complete picture of the actual sink/source present during gamma or beta oscillation, we computed the CSD maps as in Figure 2B but without prior filtering of the raw signals (Figure 8, left panels). Because sinks and sources generated by the slow oscillation are roughly constant over a single fast oscillation cycle, the main effect observed is a CSD offset, different for each electrode, and constant across the fast oscillation cycle. These offsets can be seen alone by suppressing the fast CSD modulations with a filtering of the signal in the slow frequency band (0.1–10 Hz) and then averaging across fast oscillation cycles (Figure 8, right panels). Comparing left and right panels of Figure 8, we observed that the locations of fast CSD modulations, locked to the gamma or beta cycles were not modified by the absence of low-pass filtering. However, the fast CSD modulation basal level was replaced by the actual network basal level. This should help in making hypothesis about the generation of the underlying extracellular currents.

Considering gamma oscillations (Figure 8 upper panels), in anesthetized rat, they occur close to the I/E transition (phase 0 of the slow rhythm, Buonviso et al., 2003) where excitatory input from the olfactory nerve to the GL is maximal and induces a large depolarization spread in the OB. This respectively explains the large sink offset in the GL and the source offset in most of other layers. On top of these offsets, gamma additive source in the upper GRL and sink in the upper EPL are clearly visible. This is coherent with the spike analysis (Figure 7) which explains the upper half EPL sink by a rhythmic excitation of granule cells with dendro-dendritic synapses mainly located in the upper half EPL.

Considering beta oscillations (Figure 8 lower panels), they can occur either during early or late expiration depending on the OB input (Cenier et al., 2009). This spans a large part of the slow oscillation cycle and could partially blur the offset analysis. Interestingly, slow sinks were present in the GRL (electrodes −1,−2 and −6). These sinks were confirmed by recordings from electrodes with 100-μm spacing, and were present whatever the respiratory phase (data not shown). Such sinks could originate from centrifugal excitatory activation of the GRL as expected from the strong link between beta occurrence and intact centrifugal input (Neville and Haberly, 2003; Martin et al., 2006). Taking these offsets into account, the beta current source in the GRL (shown in Figure 2B at beta phase 0, especially at electrodes −1 and −6) is actually a decrease in the slow sink offset. This effect may be due to a modulation of a centrifugal excitatory input at the beta frequency (Gao and Strowbridge, 2009). Deciphering whether beta CSD modulation comes actually from a centrifugal excitatory input or another origin like mitral rhythmic excitation (as suggested by the previous MUA analysis) cannot be done with the present data and would require additional experiments.

Overall, this shows that computing the full band CSD can bring new information to interpret the origin of the differential sublaminar profiles of beta and gamma oscillations.

## Discussion

In this study, the analysis of CSD time evolution during beta or gamma oscillation in the rat OB shows an oscillatory current sink/source dipole between the EPL and the GRL. Although this observation is in agreement with previous qualitative CSD experiments in the rat OB (Neville and Haberly, 2003), the more quantitative and detailed analysis presented here shows that there is a significant spatial shift of the sink/source dipole between both oscillation types. In particular, gamma oscillations involved the upper half of the EPL (200–300 μm from the MCL), whereas beta oscillations involved mainly the lower part of the EPL (50–150 μm from the MCL). In parallel, only the superficial part of the GRL was involved in gamma oscillation, whereas deeper granule cells were activated by beta oscillation. This shift has been demonstrated to be visible at both the group level (averaged across recording site locations) and the individual recording site level. Moreover, asymmetrical and differential activation of the OB by distinct odors did not affect gamma or beta CSD maps. Finally, the change in dipole spatial position was not a frequency effect (induced by neuron membrane low-pass filtering properties). There was a clear-cut shift between gamma and beta oscillation CSD maps, demonstrating the activation of two distinct subnetworks.

Interestingly, multi-unit analysis in the MCL showed that during both beta and gamma oscillations, the EPL current sink could be explained by mitral-to-granule excitatory synapses. Overall, the spatial shift of CSD maps can be explained by the activation of different sets of mitral excitatory synapses, segregated between the lower and upper EPL (for respectively beta and gamma oscillations), perhaps with some overlap. These would in parallel also activate, respectively, the deepest or more superficial granule cells. In addition, computing full band CSDs showed that a beta modulation of an excitatory centrifugal input could also be involved in beta oscillations generation. Thus the main result of this study is a functional demonstration of a sublaminar organization of the rat OB. Neville and Haberly (2003) did not mention such a spatial shift between the gamma and beta oscillation CSD maps. However, making a direct comparison between Neville and Haberly’s study and the present results is difficult for two main reasons. First, they only showed a single example of gamma and beta oscillation CSD and did not look at cycle-to-cycle variability with proper cycle-by-cycle averaging, which might be necessary to clearly see the shift. Second, their experimental model is different (tracheotomized animal with long and constant odor stimulations at different concentrations to generate either long gamma or long beta oscillations). Thus, without more detailed analyses of their data, the possibility that the experimental model affects the networks involved in gamma and beta oscillations in the rat OB cannot be excluded.

### Anatomical substrate for the spatial shift between beta and gamma oscillations CSD maps

Previous studies of gamma and beta oscillations in anesthetized rats have shown a remarkable stability of fast OB oscillation properties (frequency, amplitude), regardless of the odor stimulus or strength of nasal air flow (Courtiol et al., 2011; Fourcaud-Trocmé et al., 2011). The only difference between odors is their ability to evoke either one or both oscillatory regimes (Neville and Haberly, 2003; Cenier et al., 2008). Stronger OB activation generates mainly gamma oscillation, whereas weaker activation (as with alcohol in this study) generates fewer gamma and more beta oscillations. The stability of CSD maps for distinct odors, as observed here, is coherent with previous observations and suggests a strongly constrained oscillation generation mechanism. The OB network and cell properties appear to completely define fast oscillation properties, with the balance between external OB inputs (sensory or centrifugal) only selecting which oscillation is generated. This outcome may occur through a differential activation of the same set of cells, activation of distinct subnetworks in the OB or a combination of both. The present study supports the distinct subnetworks hypothesis, even if the observation that beta and gamma oscillations never occur simultaneously implies that the oscillatory subnetworks can only be activated alternatively because they are either partially overlapping or inhibiting each other. Interestingly, a previous study showed that, for a given odor, different mitral cell subsets are phase-locked to either beta or gamma oscillations (Cenier et al., 2009). This finding also supports the hypothesis of distinct subnetworks, in this case with distinct pools of mitral cells.

**Figure 9:**
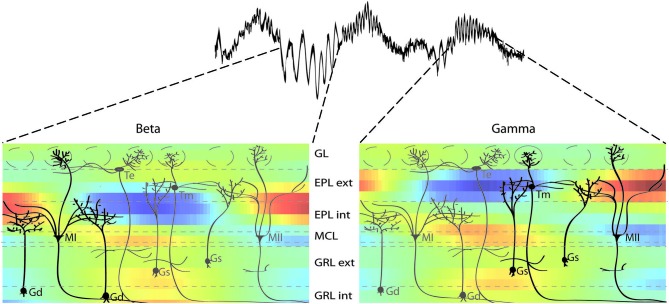
**Sublaminar network organization of the OB as revealed by functional and anatomical studies.** Combination of the different sublaminar networks involved in beta and gamma oscillations revealed by the present CSD study (color plot) and OB anatomical organization adapted from Orona et al. (1984). These data show that gamma oscillations involve mainly tufted and type II mitral cells (in black on the right panel) with more superficial secondary dendrite arborization in the external EPL. In contrast, beta oscillations involve more type I mitral cells (in black on the left panel) with secondary dendrites mainly in the internal EPL. There is a similar segregation in the GRL. Cell name abbreviations: MI: type I mitral cell; MII: type II mitral cell; Gs: superficial granule cell; Gd: deep granule cell; Te: external tufted cell; Tm: middle tufted cell.

The CSD study is only a functional and indirect demonstration of these oscillatory laminar subnetworks. The next question is whether there are anatomical substrates for these networks. Interestingly, many anatomical studies show differences between the lower and upper parts of EPL and GRL. For example, individual cell reconstructions have revealed that mitral cells can be divided into two groups, with a type I or type II group with secondary dendrites that innervate only the lower or intermediate portion of the EPL, respectively (Orona et al., 1984). The same authors demonstrated that granule cell innervation of the EPL was also inhomogeneous, with more superficial cells of the GRL innervating the upper half of EPL, and with deeper cells of the GRL innervating the deeper EPL (Orona et al., 1983). Since then, other studies have shown that anatomic or metabolic markers can display differences between the lower and upper EPL in mice or rats, including cytochrome oxidase (Mouradian and Scott, 1988) and α3-GABA subunits (Panzanelli et al., 2005; Sassoè-Pognetto et al., 2009), or between the lower and upper GRL, including perisomatic targeting granule cells (Naritsuka et al., 2009) and the functional properties and spatial distribution of newborn cells (Carleton et al., 2003; Lemasson et al., 2005). Overall, these anatomical studies provide support for a sublaminar organization of the EPL and GRL, similar to the functional observations in the present CSD study (see Figure 9 for a parallel between the anatomical substrates and the CSD profiles).

Interestingly, Wellis and Scott (1990) found that superficial granule cells in anesthetized rats responded to odor stimulation with spikes whereas deeper granule cells responded only with subthreshold depolarization. This type of physiological difference could help explain the distinct frequency range or difference between the locations of gamma and beta generators. Finally, a recent study by Manabe and Mori (2013) in awake rats suggested that the sequential activation of tufted cells and mitral cells induces high then low gamma oscillations, respectively. Due to the similar locations of secondary dendrite arborization of both type II mitral cells and tufted cells, the contributions of both cannot be distinguished in the present CSD study (see Figure 6, which shows no difference between the low and high gamma CSD maps). However, tufted cell-driven high gamma oscillation should involve the superficial EPL layer, similar to the observations for all gamma oscillations in this study.

### Relationship to fast LFP oscillations in other cortical areas and functional implications

Interestingly, in other cortical areas, recent findings have suggested that gamma activity mainly involved superficial cortical areas whereas beta oscillations mainly involved deeper layers (Roopun et al., 2008; Maier et al., 2010; Buffalo et al., 2011). Such segregation may be linked to the distinct direction of information transmission (Bastos et al., 2012, 2014; Bosman et al., 2012). These results were described in a 6-layered cortex, and the present OB results question whether such segregation exists in the OB, which is classically described as a 3-layered cortex.

From a functional point of view, OB gamma oscillations are already hypothesized as conveying feedforward information, and beta oscillations, feedback information (Buonviso et al., 2003; Neville and Haberly, 2003). In addition, beta oscillations are known to be enhanced by learning (Martin et al., 2004; Kay and Beshel, 2010) and to be disrupted by centrifugal input inactivation (Neville and Haberly, 2003; Martin et al., 2006). These results are consistent with the functional segregation of beta and gamma oscillations under investigation in other cortical areas. The sublaminar organization of the OB could thus mirror parts of the 6-layered neocortical organization and favor this functional segregation.

## Conclusion and future work

In conclusion, this study suggests a functional sublaminar organization of the rat OB supported by previous anatomical studies. This sublaminar organization could subserve distinct modulations and functions for the gamma and beta oscillations, such as their role in information transmission. This study was performed on anesthetized rats; it would be interesting to extend the study to behaving animals, in which centrifugal feedback is less depressed. A more detailed characterization of activity at the single cell level during beta or gamma oscillation will also be necessary to confirm these results. Such experiments will need to overcome some experimental limitations, such as generating beta oscillation *in vitro* or being able to have tight control of gamma vs. beta oscillation generation *in vivo*.

## Conflict of interest statement

The authors declare that the research was conducted in the absence of any commercial or financial relationships that could be construed as a potential conflict of interest.

## Footnotes

^1^Available at http://neuralensemble.org/OpenElectrophy/
